# MR Imaging of Adverse Effects and Ocular Growth Decline after Selective Intra-Arterial Chemotherapy for Retinoblastoma

**DOI:** 10.3390/cancers16101899

**Published:** 2024-05-16

**Authors:** Christiaan M. de Bloeme, Sabien van Elst, Paolo Galluzzi, Robin W. Jansen, Joeka de Haan, Sophia Göricke, Annette C. Moll, Joseph C. J. Bot, Francis L. Munier, Maja Beck-Popovic, Francesco Puccinelli, Isabelle Aerts, Theodora Hadjistilianou, Selma Sirin, Mériam Koob, Hervé J. Brisse, Liesbeth Cardoen, Philippe Maeder, Marcus C. de Jong, Pim de Graaf

**Affiliations:** 1 Cancer Center Amsterdam, Imaging and Biomarkers, 1081 HV Amsterdam, The Netherlands; 2 Department of Radiology and Nuclear Medicine, Amsterdam UMC, Vrije Universiteit Amsterdam, 1081 HV Amsterdam, The Netherlands; 3 Department of Neuroimaging Unit, Siena University Hospital, 53100 Siena, Italy; 4 Department of Diagnostic and Interventional Radiology and Neuroradiology, University Hospital Essen, 45147 Essen, Germany; 5 Department of Ophthalmology, Amsterdam UMC, Vrije Universiteit Amsterdam, 1081 HV Amsterdam, The Netherlands; 6 Unit of Pediatric Ocular Oncology, Jules-Gonin Eye Hospital, University of Lausanne, 1015 Lausanne, Switzerland; 7 Department of Pediatrics, Centre Hospitalier Universitaire Vaudois (CHUV), University of Lausanne, 1011 Lausanne, Switzerland; 8 Department of Radiology, Centre Hospitalier Universitaire Vaudois (CHUV), University of Lausanne, 1011 Lausanne, Switzerland; 9 Pediatricic Department, Institut Curie, PSL Research University, 75005 Paris, France; 10 Unit of Ophthalmology and Referral Center for Retinoblastoma, Department of Surgery, Policlinico “Santa Maria alle Scotte”, 53100 Siena, Italy; 11 Department of Diagnostic Imaging, University Children’s Hospital Zurich, University of Zurich, 8032 Zurich, Switzerland; 12 Imaging Department, Institut Curie, Paris University, 75005 Paris, France

**Keywords:** retinoblastoma, intra-arterial chemotherapy, MR imaging, adverse-effects, treatment effects

## Abstract

**Simple Summary:**

This study investigates the adverse effects of selective intra-arterial chemotherapy (SIAC) on the eyes and optic nerves of retinoblastoma patients using magnetic resonance imaging (MRI). We aim to understand the post-SIAC changes in orbital and ocular structures and evaluate their impact on eye and optic nerve growth. Experienced radiologists analyzed MR images of retinoblastoma eyes treated with SIAC, comparing them to eyes treated with other eye-saving methods and healthy eyes. Results reveal common adverse effects like inflammation and vascular changes, along with significant ocular growth arrest and optic nerve atrophy in eyes treated with SIAC, especially in children treated ≤ 12 months of age. This study underscores the importance of careful consideration when utilizing SIAC, particularly in young patients, due to its potential negative effects on eye and optic nerve development.

**Abstract:**

This retrospective multicenter study examines therapy-induced orbital and ocular MRI findings in retinoblastoma patients following selective intra-arterial chemotherapy (SIAC) and quantifies the impact of SIAC on ocular and optic nerve growth. Patients were selected based on medical chart review, with inclusion criteria requiring the availability of posttreatment MR imaging encompassing T2-weighted and T1-weighted images (pre- and post-intravenous gadolinium administration). Qualitative features and quantitative measurements were independently scored by experienced radiologists, with deep learning segmentation aiding total eye volume assessment. Eyes were categorized into three groups: eyes receiving SIAC (Rb-SIAC), eyes treated with other eye-saving methods (Rb-control), and healthy eyes. The most prevalent adverse effects post-SIAC were inflammatory and vascular features, with therapy-induced contrast enhancement observed in the intraorbital optic nerve segment in 6% of patients. Quantitative analysis revealed significant growth arrest in Rb-SIAC eyes, particularly when treatment commenced ≤ 12 months of age. Optic nerve atrophy was a significant complication in Rb-SIAC eyes. In conclusion, this study highlights the vascular and inflammatory adverse effects observed post-SIAC in retinoblastoma patients and demonstrates a negative impact on eye and optic nerve growth, particularly in children treated ≤ 12 months of age, providing crucial insights for clinical management and future research.

## 1. Introduction

Over the last two decades, the management of retinoblastoma has undergone significant changes, with a shift towards eye-preserving treatment methods and a reduction in the use of external beam radiotherapy and systemic chemotherapy [1]. One of these increasingly used therapeutic options is the targeted delivery of chemotherapeutic agents directly into the affected eye, either directly into the eye (intravitreal chemotherapy), around the eye (peri-ocular/subconjunctival chemotherapy) or selectively through the ophthalmic artery into the eye (selective intra-arterial chemotherapy [SIAC]). Compared to systemic chemotherapy, SIAC leads to higher intraocular concentrations of chemotherapy and lower systemic side effects, including reduced ototoxicity (carboplatin), neutropenia, and secondary cancers [2,3,4]. Melphalan, topotecan, and carboplatin are commonly used chemotherapeutic agents for SIAC and are delivered directly into the feeder vessels of the ocular structures [5]. Melphalan and carboplatin are alkylating agents that work by alkylating DNA and forming cross-links between DNA strands, which disrupts DNA replication and leads to cell death. Topotecan is a topoisomerase I inhibitor, an enzyme involved in DNA replication. This inhibition leads to the accumulation of DNA breaks during replication, ultimately causing cell death [6]. Multiple sessions of eye-preserving techniques are often necessary to achieve complete tumor response and preserve vision, especially in bilaterally affected patients. SIAC is an effective primary treatment for early-stage as well as for advanced-stage retinoblastoma with the benefit of preserving the eye [7,8,9,10,11,12,13,14]. For selected cases with high metastatic risk factors such as massive choroidal invasion, deep optic nerve invasion, and even intraorbital tumor infiltration, SIAC has been reported as a potential effective treatment option [15,16]. Additionally, patients with intraocular relapse after first-line treatment with SIAC can be salvaged with additional SIAC cycles [17]. Typically, two to three cycles of SIAC are sufficient for most tumors, although some tumors require up to six cycles to achieve tumor control [8,10]. It should be noted that SIAC is frequently combined with additional local therapies, such as laser photocoagulation, hyperthermia, cryotherapy, or local application of chemotherapy directly into the vitreous in patients with vitreous seeding. However, primary enucleation remains the therapy of choice in cases with metastatic risk factors, and secondary enucleation after failure of conservative treatment is sometimes necessary [18,19]. Due to these advantages and the reported therapeutic success in the literature, SIAC is increasingly used in children with retinoblastoma and has been established as a standard procedure in many centers primarily involved in retinoblastoma care.

Both SIAC and the combination of SIAC with local consolidation therapies can cause considerable toxicity to normal ocular tissues [20]. Despite the benefits of SIAC, treatment-related side effects occur and can have a detrimental effect on the visual potential. Intraocular complications, such as chorioretinal atrophy (4%), choroidal occlusive vasculopathy (choroidal infarction) (8%), occlusion of the ophthalmic artery, central retinal artery or central retinal vein (up to 15%), vitreous hemorrhage (2%), retinal detachment (8%), and optic nerve atrophy (8%) are of serious concern [9,18,19,21,22,23]. Other frequent (peri)orbital side effects include eyelid edema (11%), loss of eyelashes (13%), ptosis (6%), and ophthalmoplegia (8%), generally resolving within 2–3 months [24,25]. Most of the reported side effects are identified through routine clinical, ophthalmologic, and fundoscopic examinations. However, potential intraorbital side effects cannot be depicted without cross-sectional imaging.

Pretreatment MRI is routinely performed to provide insights into the size, location, and potential spread of the tumor. However, limited information is available regarding the posttreatment imaging appearance of treatment effects following SIAC. This is primarily because posttreatment (including post-SIAC) MR imaging is typically reserved for patients with an opaque ocular media (i.e., vitreous hemorrhage or cataract) hindering fundoscopy and is not routinely performed. MR imaging is also conducted in instances of suspected intraorbital complications, for monitoring tumors in proximity to or covering the optic nerve to rule out post-laminar optic nerve infiltration, and (in some centers) for screening on retinoblastoma associated midline brain tumors (trilateral retinoblastoma). Currently, only one series of 60 patients has been published, showing MR findings of some intraocular and orbital complications and vascular events, as well as a reduced size of the SIAC-treated eye compared to pretreatment imaging in 67% of patients [26]. To further explore the spectrum of potential imaging findings that can be encountered by radiologists on MRI after SIAC-based eye-salvage treatment, a retrospective multicenter study with a considerable number of patients was initiated. Therefore, the purpose of this study is to provide an extensive overview of therapy-induced orbital and ocular MR imaging findings and adverse effects after SIAC for retinoblastoma and to quantitatively assess the effect of the therapy on eye growth.

## 2. Materials and Methods

### 2.1. Study Design and Patient Selection

This study was a non-consecutive, multicenter retrospective case series. Patients treated with SIAC in retinoblastoma referral centers within the European Retinoblastoma Imaging Collaboration (ERIC) were eligible for inclusion. Medical chart review was used to select patients, and patients were included when MR images obtained after initiation of SIAC treatment were available containing at least T2-weighted and T1-weighted images (before and after intravenous gadolinium administration). In participating centers, posttreatment MRI was not performed as part of a routine clinical protocol but only on indication (i.e., evaluation of clinical side-effects after SIAC, treatment response evaluation, or trilateral retinoblastoma screening). Pretreatment MR imaging studies were collected when available. In patients with bilateral disease, both eyes were included, and independence of each eye was assumed during further analysis.

Eyes of the included SIAC patients were divided into three main groups of interest: retinoblastoma eyes that were treated with SIAC (Rb-SIAC), retinoblastoma eyes that were treated with other eye-preserving treatment than SIAC (Rb-control), and contralateral unaffected healthy eyes (healthy-controls).

Additionally, MR imaging studies of unilaterally affected retinoblastoma patients treated with primary enucleation were included for the quantitative eye volume measurement, as their contralateral unaffected eye acted as a supplement to the healthy-controls. The enucleated eye was excluded from the study.

### 2.2. Qualitative Image Analysis

Four experienced radiologists (experience ranged from 18 to 33 years in ocular MR imaging) from the ERIC group independently evaluated MR images of SIAC eyes, blinded for clinical data. Imaging features included items from the validated “Retinoblastoma Imaging Atlas” [27], supplemented with features of known clinical adverse effects, selected after the literature review [18,19,25] and expert discussion, see Table S1. Thereafter, a centralized review of imaging with abnormal results was performed during a meeting with all radiologists to reach a consensus.

### 2.3. Quantitative Image Analysis

Each radiologist performed two quantitative measurements on available axial MR images. Optic nerve width at 3 mm posterior to the lamina cribrosa was measured to evaluate optic nerve atrophy, and axial eye length was measured to evaluate eye growth in accordance with the previously published method [28].

After quantitative measurements were performed by radiologists in each participating center, a centralized measurement of eye volume was obtained through a previously published automatic segmentation using a Multi-View Convolutional Neural Network (MV-CNN) [29]. Only scans that were digitally transferred for central analysis could be included in these measurements. To handle the multicenter data in this study, the MV-CNN was retrained on manual delineations from all included centers. Manual delineation of eye volume was performed on contrast-enhanced T1-weighted MR images by an independent reviewer (C.M.d.B. with 5 years of experience in ocular MR imaging) using 3D Slicer (Version 4.10.1, MIT, USA) and were validated by expert radiologists (P.d.G. and M.C.d.J. with respectively 18 and 11 years of experience in ocular imaging).

All automatic segmentations made by the deep learning network were visually checked by C.M.d.B. for accuracy and adjusted if they were visually inadequate. Total eye volume from the segmentations was calculated by counting the number of pixels multiplied by the pixel dimensions.

### 2.4. Statistical Analysis

The Mann-Whitney *U*-test was used to compare continuous data, and the Fisher Exact test to compare dichotomous data. For the qualitative scored features, the frequency was determined by the stage of the scan (pretreatment or posttreatment). If a patient had multiple scans at a stage, only the first scan of that stage was included in the analysis, and the other scans were classified as follow-up scans.

For the analysis of the quantitative measurements, the three main groups (Rb-SIAC, Rb-controls, and healthy-controls) were further divided into a pretreatment group and a posttreatment group. Radiologists’ quantitative measurements were analyzed by comparing the main groups with each other using the Mann-Whitney *U*-test. The pretreatment and posttreatment groups were compared using the Wilcoxon signed-rank test. Additionally, eye volumes of each subgroup were compared with multivariable forward linear regression analysis with predictors of age, gender, center, and main group of interest. The predictor center of inclusion might be biased due to the supplement of the healthy-controls from the contralateral eyes of the unilateral enucleated retinoblastoma patients.

To calculate the effect of age at the start of SIAC on subsequent eye growth arrest, the three main groups (Rb-SIAC, Rb-controls, and healthy-controls) of interest were used again. Rb-SIAC was divided into groups of eyes that were treated with SIAC before or at (≤) and after (>) 12 months of age. The groups were compared to each other with a multivariable forward logistic regression analysis with predictors of age at scan, gender, and eye volume. Eyes were not allowed to enter twice in this analysis. Thus, if both pretreatment and posttreatment scans were available, the posttreatment scan was selected. All the statistical calculations were performed using SPSS software (version 28; SPSS, Chicago, IL, USA).

## 3. Results

### 3.1. Patients

**Figure 1:**
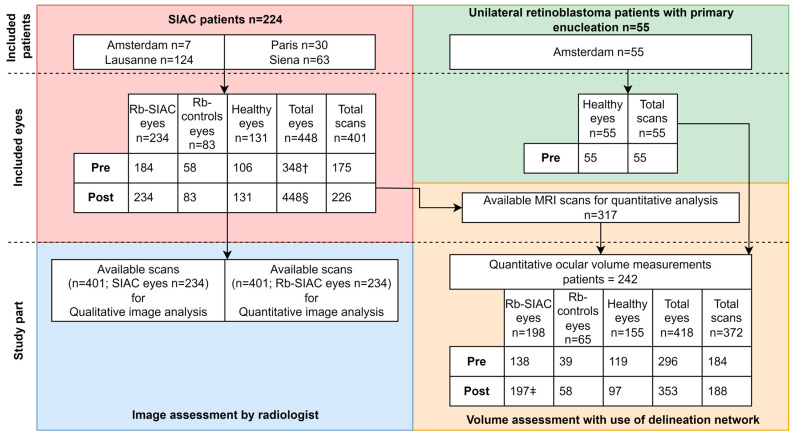
Patient inclusion flowchart. Note.- Data are presented as the number of eyes, number of scans, or number of patients. SIAC = selective intra-arterial chemotherapy, Pre = pretreatment scan, Post = posttreatment scan. † Total number of eyes is lower than expected (scans times two), because one patient who underwent SIAC in both eyes only had a pretreatment scan for one of its eyes and another SIAC patient who underwent SIAC in both eyes had a pretreatment scan for each eye individually. § Two patients had a posttreatment scan for each individual eye, making the number of posttreatment eyes (448) less than twice the number of scans (226). ǂ Patient who underwent SIAC in both eyes of whom one eye was enucleated before the posttreatment scan.

A total of 224 patients were treated with SIAC, and an additional 55 unilateral retinoblastoma patients who underwent primary enucleation were included. Of the enucleated unilateral retinoblastoma patients, only the healthy contralateral eye was included (flowchart, see Figure 1). Of the 224 SIAC patients, 10 were treated with SIAC treatment in both eyes. After the application of the selection criteria, the study included a total of 456 MRI scans, including 55 pretreatment scans from the primary enucleation patients (healthy-controls), 175 baseline scans from SIAC patients prior to treatment, and 226 scans posttreatment. Fourteen patients had multiple follow-up scans after their posttreatment scan with a median of 2 scans per patient (interquartile range [IQR]: 1–4 scans; range: 1–6 scans; 39 scans in total). Patient inclusion and patient characteristics of the dataset are summarized in Figure 1 and Table 1, respectively. A portion of the SIAC patients has been previously published in an article that focused on acute choroidal ischemia after SIAC [30]. For the included Rb-SIAC eyes, the majority were classified as D according to the International Classification of Retinoblastoma (ICRB) (149 eyes out of 234 [64%]). The majority (n = 153; 65%) of RB-SIAC eyes were treated with a single drug (Melphalan). The median number of SIAC cycles was 3 (IQR: 2–5; range: 1–9). The median time between the last SIAC cycle and posttreatment scan was 3 months (IQR: 1–11; range: 0–95). Clinical parameters for the individual eyes that were treated with SIAC are summarized in Table 2.

### 3.2. Qualitative Imaging Features

**Figure 2:**
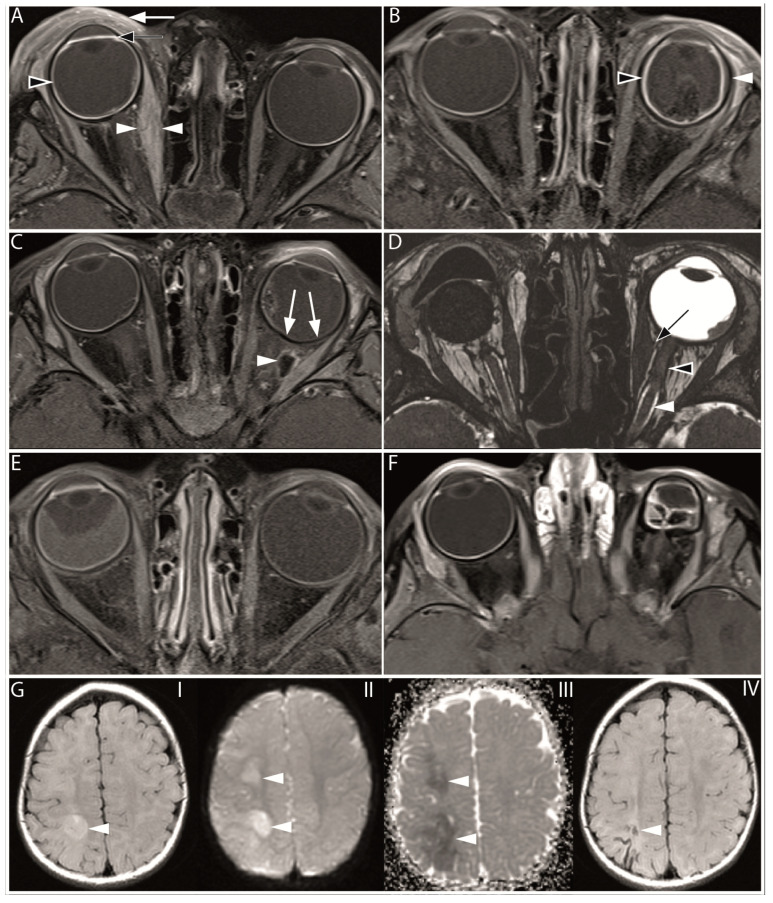
Overview of MR imaging features found after selective intra-arterial chemotherapy. (**A**) Axial contrast-enhanced T1-weighted MR image with fat suppression of a 29-month-old patient, 5 days after the last SIAC course (one cycle of 8 mg melphalan and one cycle of 2 mg topotecan). The image reveals swelling and increased enhancement of the preseptal soft tissue, indicative of palpebral inflammation (indicated by the white arrow). Furthermore, there is increased enhancement of the anterior eye segment (marked by the black arrow) and increased enhancement and choroidal thickening (indicated by the black arrowhead). Also, there is swelling and increased enhancement of the medial rectus muscle (white arrowheads), suggesting extraocular muscle inflammation. (**B**) Axial contrast-enhanced T1-weighted MR image with fat suppression of a 13-month-old patient, 1 month after the last SIAC course (a total of six cycles with a cumulative dose of 19 mg melphalan and two cycles with a cumulative dose of 4 mg topotecan [2 mg per cycle]). The choroid shows increased enhancement and thickening (black arrowhead), and on the lateral size, the choroid is detached from the sclera, indicative of choroidal effusion (white arrowhead). (**C**) Axial contrast-enhanced T1-weighted MR image with fat suppression of a 17-month-old patient, 1 month after the last SIAC course of the left eye (a total of two cycles with a cumulative dose of 8 mg melphalan [4 mg per cycle]). A large area of the choroid shows decreased contrast enhancement, indicative of choroidal infarction (white arrows). Additionally, a large hypointense area with a hyperintense border in the intraorbital fat is indicative of orbital fat necrosis (white arrowhead). Both choroidal infarction and fat necrosis were not present on the baseline scan (baseline scan is not shown). (**D**) Axial 3D T2-weighted MR image sequence of a 127-month-old patient, 17 months after the last SIAC course of the left eye (a total of two cycles with a cumulative dose of 8 mg melphalan [4 mg per cycle]). The right eye was enucleated. The proximal optic nerve has a normal signal intensity surrounded by the bright signal of the cerebral spinal fluid (CSF) (white arrowhead). In the distal part of the optic nerve, this CSF signal (black arrowhead) is absent, indicating perineural optic nerve fibrosis. Additionally, the medial extraocular muscle shows some traction towards the optic nerve, which is suggestive of ocular muscle fibrosis (black arrow). (**E**) Axial contrast-enhanced T1-weighted MR image with fat suppression of a 22-month-old patient on the same day as the last SIAC cycle (a total of two cycles with a cumulative dose of 8 mg melphalan [4 mg per cycle]). The right eye is smaller than the left eye, possibly caused by an arrested growth. Also, total retinal detachment, slightly increased enhancement and thickening of the choroid, and increased enhancement of the anterior eye chamber can be seen. (**F**) Axial contrast-enhanced T1-weighted MR image with fat suppression of a 12-month-old patient on the same day as the last SIAC cycle (a total of two cycles with a cumulative dose of 5 mg melphalan). The first SIAC cycle was complicated by the wedging of the ophthalmic artery by the catheter. The left eye shows phthisis bulbi. (**G**) Axial MR images of a 10-months-old patient with an acute cerebral infarction (white arrowhead), three days after an unsuccessful second SIAC cycle through the ophthalmic artery (a total of two cycles with a cumulative dose of 4.7 mg melphalan [first a cycle of 4 mg, followed by an additional 0.7 mg]). The second SIAC cycle was aborted after infusion of about 1/6th of the intended dose (4 mg) due to a systemic adverse reaction to the contrast agent. The fluid-attenuated inversion recovery MR image (I) shows an area of bright signal intensity in the right hemisphere, which is confirmed by the diffusion-weighted imaging (II) and apparent diffusion coefficient images (III) showing areas of restricted diffusion, indicative of acute cerebral ischemia. After three months, a follow-up fluid-attenuated inversion recovery image (IV) showed a small area of tissue loss in the right parietal lobe.

A total of 15 qualitative imaging features were scored and are summarized in Table 3, and some features are illustrated in Figure 2.

Inflammatory features were frequently present on posttreatment scans (Table 3). The most common intraorbital inflammatory signs were swelling and increased enhancement of the extraocular muscles (myositis), which was detected on posttreatment scans in 29% of patients, followed by the presence of orbital fat enhancement (cellulitis) in 19%. Uncommon intraorbital findings on posttreatment scans were extraocular muscle fibrosis (<1%), fibrotic changes around the optic nerve (<1%), and orbital fat necrosis (1%). Intraocular imaging findings consisted of choroidal thickening and increased enhancement in 27% and enhancement of the anterior eye segment (24%). Focal absence of choroidal enhancement as a sign of choroidal infarction was present in 10% of the posttreatment scans. Although vitreous hemorrhage was a relatively uncommon finding (2%), the incidence of subretinal hemorrhage was much higher (16%). Retinal detachment is commonly associated with retinoblastoma and had a prevalence of 47% (95% CI: 38–58) on pretreatment scans, which decreased to 35% (95% CI: 29–42) on posttreatment scans. Preseptal orbital cellulitis with eyelid swelling and fat enhancement was present in 13% of posttreatment scans. Out of 224 patients that were treated with SIAC (in total 974 procedures), 3 patients (1%, 95% CI: 0–3) had an ischemic cerebrovascular accident (Figure 2).

**Figure 3:**
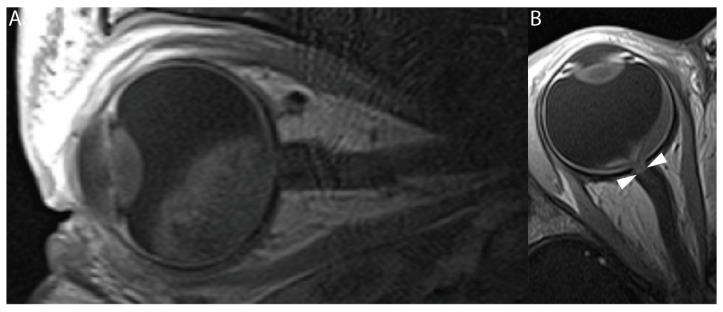
Baseline and posttreatment T1-weighted contrast-enhanced MRI of a unilateral retinoblastoma patient. (**A**) Sagittal contrast-enhanced T1-weighted MR image of a 13-month-old patient with unilateral retinoblastoma. This pretreatment image does not show an increased enhancement of the distal optic nerve. (**B**) Axial contrast-enhanced T1-weighted MR image of the same patient performed after the last SIAC course (21 months of age). The patient received a total of six cycles of combined 24 mg melphalan (4 mg per cycle) and 6 mg of topotecan (1 mg per cycle). Increased contrast enhancement can be seen in the most distal part of the optic nerve (white arrowheads). After enucleation, tumor invasion was ruled out, suggesting that the increased enhancement of the optic nerve was most likely caused by an SIAC-induced inflammatory event.

**Figure 4:**
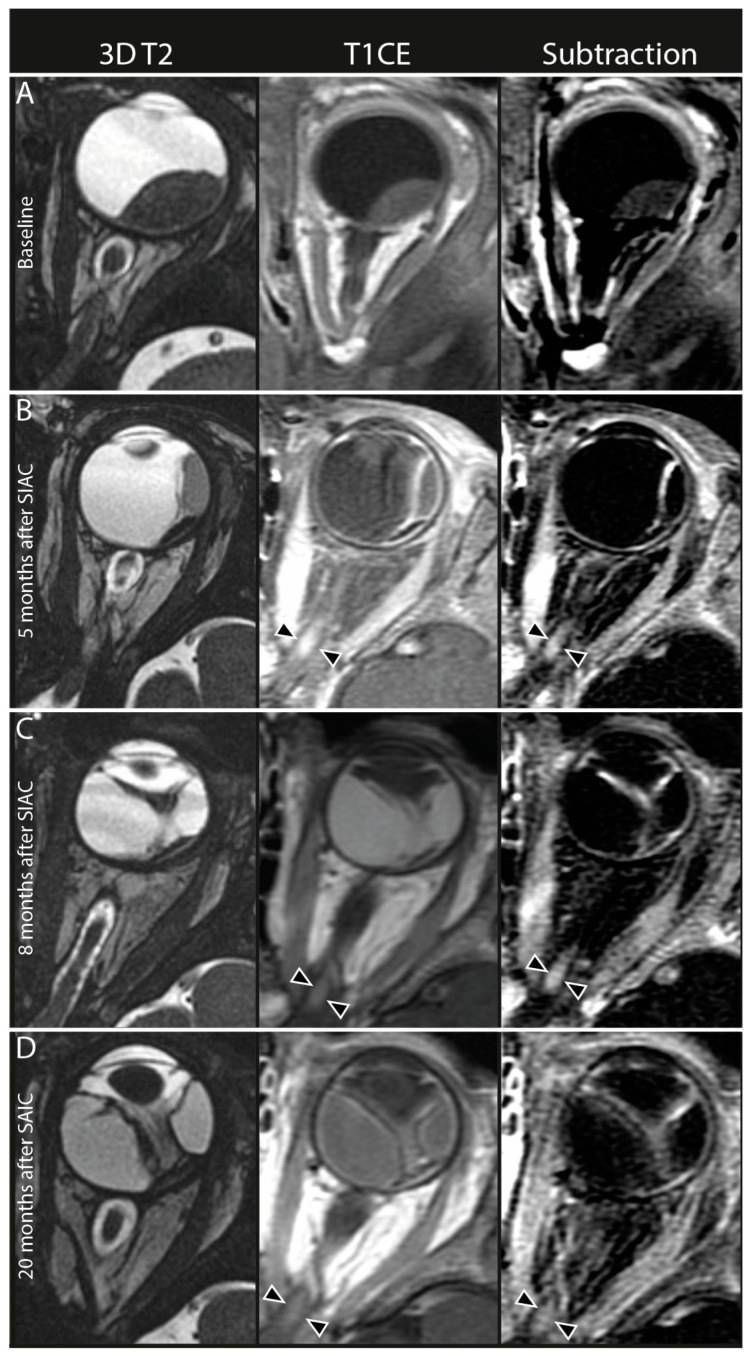
Persistent optic nerve enhancement after selective intra-arterial chemotherapy (SIAC). In the left column, axial 3D T2-weighted MR images (3D T2), middle column axial contrast-enhanced T1-weighted MR images (T1CE), and in the right column axial subtraction images from contrast-enhanced T1-weighted MR images minus native T1-weighted MR images (subtraction). (**A**) Pretreatment MRI scan showed no optic nerve enhancement in any part of the optic nerve. (**B**) First follow-up MRI 5 months after the last SIAC cycle (a total of three cycles of combined 10 mg melphalan [3 mg for the first and second cycle, and 4 mg for the last cycle]. The procedure was conducted through the carotid artery with the catheter tip in the ostium of the ophthalmic artery with no wedging or complications. Enhancement of the proximal part of the optic nerve (black arrowheads). Persistent enhancement of the optic nerve 8 (**C**) and 20 (**D**) months after the last SIAC cycle (black arrowheads).

**Figure 5:**
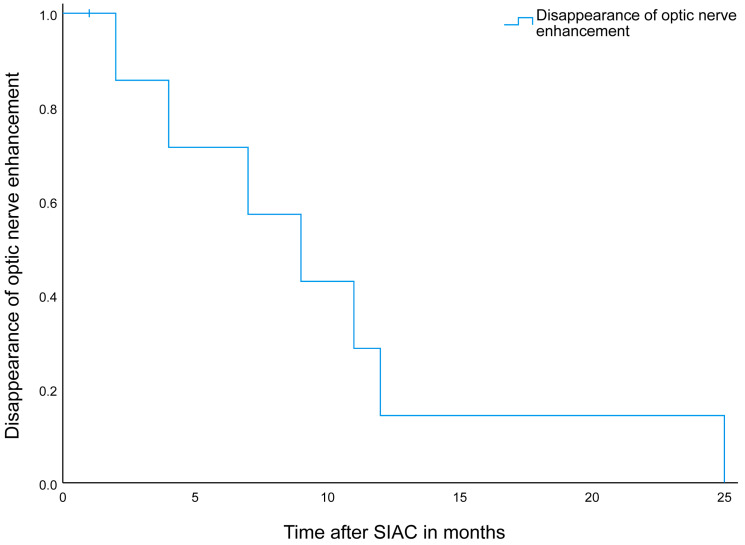
Disappearance of optic nerve enhancement after selective intra-internal chemotherapy on follow-up MR imaging. Follow-up of the optic nerve enhancement after selective intra-arterial chemotherapy in nine patients with a median persistence of 9 months (95% CI 4–14 months). Note.- SIAC = selective intra-internal chemotherapy.

New enhancement of the intraorbital part of the optic nerve was detected on posttreatment scans in 15 out of 233 eyes (6.4%, 95% CI: 4–10). None of these patients showed optic nerve enhancement before the start of SIAC treatment. This post-SIAC optic nerve enhancement was detected in the entire intraorbital part of the optic nerve from the most distal (post-laminar) (Figure 3) to the proximal part (near the optic canal) (Figure 4). In nine of these patients, follow-up scans were performed, and the enhancement persisted during a median of 9 months (95% CI 4–14 months) after the last SIAC (Figure 5). Enucleation was performed in only two eyes (13.3%, 95% CI: 2–40).

### 3.3. Quantitative Image Analysis

The optic nerve of Rb-SIAC eyes showed a statistically significant size reduction with a mean diameter of 2.76 mm on the pretreatment scan versus 2.71 mm on the posttreatment scan (*p* < 0.001, Wilcoxon signed-rank test). In contrast, Rb-controls and healthy-controls had significant increase in mean optic nerve diameter (pretreatment 2.74 mm versus posttreatment 2.87 mm, *p* = 0.006 and pretreatment 2.87 mm versus posttreatment 2.98 mm, *p* < 0.001, respectively) (Table S2).

The mean optic nerve diameter difference on the post- and pretreatment scan between Rb-SIAC (−0.11 mm) and Rb-controls (0.09 mm) and between Rb-SIAC and healthy-controls (0.07 mm) were both statistically significant (*p* < 0.001), whilst healthy-controls and Rb-controls were not (*p* = 0.55) (Table S2).

On the pretreatment scans, there was no statistically significant difference between the mean axial length of Rb-SIAC (20.31 mm) and Rb-controls (20.37 mm) (*p* = 0.43). However, both groups of retinoblastoma eyes were significantly smaller compared to the healthy-controls (20.98 mm) at baseline (Table S2). This difference remained statistically significant on the posttreatment scans. After treatment, Rb-SIAC eyes (20.49 mm) were significantly smaller than Rb-controls (21.11 mm) (*p* = 0.001) and healthy-controls (21.53 mm) (*p* < 0.001). Also, there was less increase in the mean difference of the axial length between the pretreatment versus posttreatment scans (delta of the axial length) in Rb-SIAC (0.12 mm) versus Rb-controls (0.71 mm) (*p* < 0.001) and Rb-SIAC and healthy-controls (0.50 mm) (*p* < 0.001). There was no significant difference in the delta of the axial length when comparing healthy-controls versus Rb-controls.

### 3.4. Deep Learning Delineation Network

Eighty-four eyes on contrast-enhanced T1-weighted images originating from Lausanne, Siena, and Amsterdam were manually delineated for training and validation purposes of the deep learning segmentation network. The MV-CNN obtained a mean Dice Similarity Coefficient (DSC) of 0.95 with a standard deviation of 0.014 and an intraclass correlation coefficient (ICC) of 0.97 during training. Subsequently, the trained MV-CNN was used to automatically segment all available contrast-enhanced T1-weighted images. These segmentations were used to quantify the volume of the eye globes in each patient. For a more detailed explanation and results, see Supplement A, Figure S1 and Table S3.

### 3.5. Quantitative Ocular Volume Measurements

A total of 242 patients with 372 scans (184 pretreatment and 188 posttreatment scans) were included in quantitative analysis using the MV-CNN, Figure 1. The median number of SIAC cycles was the same for eyes that were treated with SIAC ≤ 12 months of age versus eyes that were treated with SIAC > 12 months of age (*p* = 0.75, Mann-Whitney *U*-test). Characteristics for each group are summarized in Table S4. Retinoblastoma eyes (Rb-controls and Rb-SIAC) had a statistically significantly smaller eye volume on pretreatment scans compared to healthy-controls (*p* ≤ 0.001, corrected for age and center). Eye volume on the pretreatment scan was significantly smaller for Rb-SIAC in comparison to Rb-controls (*p* = 0.039, corrected for age and center). On posttreatment scans, the healthy-controls did not significantly differ from Rb-controls (*p* = 0.062 corrected for age). However, healthy-controls did show a larger eye volume on the posttreatment scan compared to Rb-SIAC eyes (*p* < 0.001, corrected for age). Also, Rb-SIAC eyes were significantly smaller than Rb-controls (*p* < 0.001, corrected for age). Multivariable linear regression models are shown in Table S5. Individual volume changes per eye before and after treatment for the healthy-controls, Rb-controls, and Rb-SIAC are shown in Figure S2.

### 3.6. The Effect of Age at the Start of SIAC on Subsequent Eye Growth Arrest

**Figure 6:**
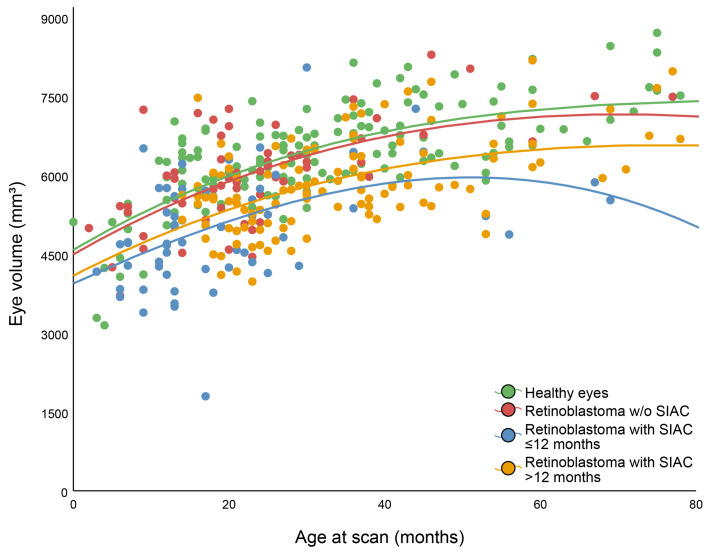
Eye volume as a function of age at scan in months for four different groups. Note.- Lines created using the fit method cubic.

The multivariable logistic regression with its predictors and *p* values are shown in Table 4 and Figure 6. Eye volume was a significant predictor to differentiate between healthy-controls or retinoblastoma controls and eyes that were treated with SIAC ≤ 12 months of age (*p* < 0.001 and *p* < 0.001, respectively). For the groups of healthy-controls and eyes that were treated with SIAC > 12 months of age, age at scan and eye volume were both predictors to differentiate between groups (*p* < 0.001 and *p* < 0.001, respectively). Moreover, predictors age at scan and eye volume were also both able to differentiate between Rb-controls and eyes that were treated with SIAC > 12 months of age (*p* < 0.001 and *p* = 0.007, respectively).

When comparing eyes that were treated with SIAC ≤ 12 months of age and eyes that were treated with SIAC > 12 months of age, the number of SIAC cycles was added as an additional predictor. However, this predictor did not exhibit statistical significance (*p* = 0.06), while age at scan and volume were (*p* < 0.001 and *p* < 0.001, respectively).

## 4. Discussion

This multicenter study provides an overview of the therapy-induced orbital and ocular MR imaging findings after SIAC for retinoblastoma. After SIAC, inflammatory features and vascular complications were most prevalent, while new areas of contrast enhancement anywhere within the intraorbital segment of the optic nerve were also encountered. Findings from quantitative analysis suggest that SIAC-treated eyes, especially those that were treated ≤12 months of age, had a significant growth arrest compared to Rb-controls and healthy-controls. Also, optic nerve atrophy is a significant complication in retinoblastoma eyes after SIAC.

Our qualitative analysis of posttreatment MRI scans revealed that intraocular and orbital vascular complications and inflammatory features were frequently present after SIAC. SIAC can cause various intraocular irreversible and potentially vision-threatening adverse effects of which choroidal occlusive vasculopathy or choroidal ischemia is commonly reported [22]. On MRI, focal absence of choroidal enhancement as a sign of choroidal infarcts was present in 10% of the posttreatment scans in this study, which is in line with the literature [26]. Increased leakage of contrast from the structures of the uvea can be a result of vascular damage or inflammation, which may be attributed to the toxicity of the drugs to the vascular bed [31,32]. Posttreatment extraocular tissue swelling and increased enhancement were also present in other orbital compartments. Eyelid edema is a well-known and clinically easily detectable side-effect of SIAC and is seen in 10–15% of infusions [33]. Swelling of the preseptal orbital structures in combination with fat enhancement (cellulitis) was detected in 13% of posttreatment scans. Furthermore, these inflammatory signs were also frequently encountered in a significant number of patients in the intraorbital compartments that cannot be evaluated directly during routine clinical examination. These imaging features have the potential to serve as early predictors for clinical symptoms and elucidate certain clinical presentations. For instance, extraocular muscle inflammation with increased enhancement and swelling of the muscle is seen on almost a third (29%) of posttreatment MRI scans and can lead to ophthalmoplegia or strabismus. Orbital fat enhancement (cellulitis) was also a common finding (19%) after SIAC and can lead to clinically detectable proptosis. In contrast, a decrease in the amount of orbital fat has been described before, where a 20% fat volume reduction was measured 18 months after SIAC compared with baseline [34]. In our series, we encountered 2 cases of orbital fat necrosis. Quantification of the amount of fat tissue was not performed in this study. However, it can hypothesized that the reactive orbital cellulitis or even fat necrosis after SIAC could be an important indicator for subsequent fat volume reduction during follow-up.

In our study, we observed new focal areas of contrast enhancement in the orbital segment of the optic nerve in 6% of patients after SIAC. This was encountered in all parts of the intraorbital segment of the nerve, including the most distal (post-laminar) part of the nerve, which can mimic optic nerve tumor invasion. Inflammatory contrast enhancement in the distal part of the nerve mimicking tumor invasion was described before in the context of extensive tumor necrosis with associated orbital cellulitis [35]. A subgroup of our patients with newly developed optic nerve enhancement after SIAC had additional follow-up scans after the first posttreatment scan. A gradual decrease in contrast enhancement occurred over the course of months (Figure 5), although in one patient, the enhancement persisted for up to 25 months. Tumor invasion of the optic nerve was eventually ruled out by either extended follow-up imaging or by histopathologic analysis after enucleation. It is known that reactive enhancement can be appreciated after optic nerve damage, for instance, at the cut-end of the optic nerve after enucleation, where it also shows a gradual disappearance over time [36].

Another adverse effect was optic nerve atrophy, which can be a significant complication in retinoblastoma eyes after SIAC. Optic nerve atrophy can be detected on MRI scans. Quantitative image analysis showed a decrease in optic nerve diameter after SIAC in this study, whereas a mean diameter increase was measured in the Rb-controls and healthy-controls subgroups representing normal optic nerve growth. Furthermore, it must be noted that before treatment initiation, there was no difference between the mean optic nerve diameter between the Rb-SIAC and Rb-controls, although both subgroups already showed a significantly smaller mean optic nerve diameter compared to healthy-control eyes. Therefore, it is important to mention that not only the treatment but also the presence of retinoblastoma itself can induce optic nerve atrophy. This atrophy is caused by the degeneration of retinal ganglion cells, which leads to the direct destruction of the retina by the tumor or by long-standing retinal detachment. Optic nerve atrophy is associated with loss of visual acuity, and early recognition may be clinically relevant for managing patients with retinoblastoma who are treated with SIAC.

We hypothesize that both optic nerve enhancement and optic nerve atrophy have a common cause, the local application of a relatively high dosage of chemotherapy through the ophthalmic artery. This may cause regional vascular toxicity, leading to inflammatory pathological changes in the small blood vessels supplying the optic nerve, resulting in contrast enhancement of the optic nerve itself. These pathological changes could lead to thrombosis, leukostasis, and occlusion of these vessels [31]. Evidence from a porcine model supports our hypothesis. In this model, when topotecan was administered via ophthalmic artery catheterization, drug concentrations in the proximal and distal portions of the optic nerve were found to be 80 times higher compared to intravenous infusion [37]. These concentrations greatly exceeded the IC50 (half-maximal inhibitory concentration) of topotecan, indicating that the drug reached potentially toxic levels in the optic nerve tissue. Therefore, our findings suggest that the focal enhancement observed in the optic nerve after SIAC may be attributed to regional vascular toxicity, inflammation, or even irreversible nerve damage. Further research is needed to fully understand the mechanisms and clinical implications of this phenomenon.

The quantitative analysis of axial eye length and ocular volume revealed that the eyes of patients with retinoblastoma, both those who were treated with SIAC and those who did not, had a smaller eye size compared to healthy-control eyes. This difference was present on pretreatment scans as well as on the posttreatment imaging. This suggests that the presence of retinoblastoma itself may contribute to growth retardation of the eye, which is in line with an earlier study [28]. However, after SIAC treatment, eyes that were treated with treatment ≤ 12 months of age had a significant growth arrest compared to those that were treated >12 months of age and the Rb-controls group. This indicates that the effect of SIAC on growth arrest is more significant than the effect of the presence of retinoblastoma alone. Furthermore, this effect seems to be regardless of the number of SIAC cycles received. This growth arrest might also be explained by the above-mentioned hypothesis.

Three patients (1%) experienced periprocedural intracerebral complications, of whom two patients were already described in a former publication [30]. The additional patient presented with seizures three days after a right-sided catheterization of the ophthalmic artery. The procedure was complicated by severe episodes of bradycardia and hypotension during selective ophthalmic artery catheterization and, therefore, terminated without successful delivery of melphalan [30]. MRI showed diffuse right-sided intracerebral areas of ischemia in the right middle cerebral artery territory. The patient completely recovered without residual long-term neurological symptoms.

### Limitations

This study has several limitations. It was a retrospective study, and despite being a relatively large cohort, the cohort size may still be limited for certain features. Moreover, all the imaging features were scored by a single reader followed by a consensus reading of the imaging features, while separate scoring by multiple readers would have been preferred to limit bias and to assess inter-reader agreement. A potential source of selection bias is the increased likeliness that patients presenting with symptoms were more likely to receive an additional MRI scan, potentially resulting in higher occurrences of adverse effects. Furthermore, clinical protocols for imaging in patients after SIAC were not uniform in the included centers. Most centers only performed posttreatment imaging on indication and not as part of a routine clinical protocol. Also, treatment regimens and indications for SIAC might differ for each center.

## 5. Conclusions

In conclusion, this study provides a retinoblastoma imaging reference for adverse effects after selective intra-arterial chemotherapy. Notably, selective intra-arterial chemotherapy negatively impacted eye and optic nerve growth, especially in children that were treated ≤ twelve months of age.

## Figures and Tables

**Table 1:** Clinical parameters for the included patients.

Group	SIAC: n = 224 Patients (80%)	Healthy-Controls: n = 55 Patients (20%)	Total: n = 279 Patients
Age at first SIAC in months; median [IQR], (range)	18 [11–31], (4–171)	n/a	18 [11–31], (2–171)
Age at pretreatment MRI scan in months; median, [IQR], (range)	16 [9–31], (1–169 *)	27 [16–45], 0–113	16 [9–33], (1–169 *)
Age at posttreatment MRI scan in months; median, [IQR], (range)	30 [19–47], (8–181 *)	n/a	30 [19–47], (8–181 *)
**Gender**			
Female n, (%)	95 (42)	25 (45)	120 (43)
Male n, (%)	129 (58)	30 (55)	159 (57)
**Laterality**			
Unilateral n, (%)	131 (59)	55 (100)	186 (67)
Bilateral n, (%)	93 (42)	n/a	93 (33)
**Treated eyes**			
OD n, (%)	114 (51)	25 (45)	139 (50)
OS n, (%)	100 (45)	30 (55)	130 (47)
ODS n, (%)	10 (4)	n/a	10 (4)

Note.—Data presented age in months; median, [interquartile range], (range) or as number of patients with percentages in parentheses or as number of eyes with percentages in parentheses. The total percentages might be lower or higher than 100 due to rounding of the numbers. SIAC = selective intra-arterial chemotherapy. n/a = not applicable. * calculated only on available scans.

**Table 2:** Clinical parameters for the included eyes treated with selective intra-arterial chemotherapy.

Number of Eyes Treated with SIAC †	n = 234
**ICRB**	
A n, (%)	1 (<1)
B n, (%)	25 (11)
C n, (%)	19 (8)
D n, (%)	149 (64)
E n, (%)	31 (13)
Missing	9 (4)
**Types of drugs**	
Melphalan only n, (%)	153 (65)
Melphalan and topotecan * n, (%)	64 (27)
Melphalan and carboplatin n, (%)	2 (<1)
Melphalan, topotecan and carboplatin n, (%)	15 (6)
**SIAC**	
Number of SIAC cycles; median [IQR], range	3, [2–5], (1–9)
Months between last SIAC cycle and posttreatment MRI scan; median, [IQR], (range)	3 [1–11], (0–95)

Note.—Data presented as numbers with percentages in parentheses of eyes or age in months median [interquartile range], (range) or number of SIAC cycles; median, [interquartile range], (range) or months between last SIAC cycle and posttreatment MRI scan; median, [interquartile range], (range). The total percentages might be lower or higher than 100 due to rounding of the numbers. SIAC = selective intra-arterial chemotherapy. * One bilateral patient was treated with SIAC in both eyes, alternating between melphalan and topotecan. † Both eyes of patients bilaterally treated with selective intra-arterial chemotherapy were included.

**Table 3:** Prevalence of features in eyes treated with selective intra-arterial chemotherapy on pre- and posttreatment MRI scans.

Scanning Time	Pretreatment MRI	Posttreatment MRI
**Feature**	n of events/n of eyes, % (95% CI)	n of events/n of eyes, % (95% CI)
**Ocular**		
Retinal detachment	87/184, 47 (38–58)	82/234, 35 (29–42)
Subretinal hemorrhage	43/182, 24 (17–31)	37/234, 16 (11–21)
Vitreous hemorrhage	3/183, 2 (0–5)	5/234, 2 (1–4)
**Enhancement of the anterior eye segment**	n/a	55/231, 24 (18–30)
Choroidal infarction	n/a	24/233, 10 (7–15)
Choroidal thickening	n/a	62/234, 27 (21–33)
**Orbital (Preseptal space)**	n/a	
Palpebral inflammation (cellulitis)	n/a	29/231, 13 (9–18)
**Orbital (Postseptal space)**	n/a	
Extraocular muscle fibrosis	n/a	1/234, 0 (0–2)
Extraocular muscle inflammation (myositis)	n/a	68/233, 29 (23–35)
Orbital fat enhancement (cellulitis)	n/a	41/232, 18 (13–24)
Orbital fat necrosis	n/a	2/232, 1 (0–7)
Optic nerve enhancement	n/a	15/233, 6 (4–10)
Perineural optic nerve fibrosis	n/a	1/226, 0 (0–2)
**Intracranial**		
Cerebral infarction	n/a	3/234, 1 (0–4)

Note.—Data presented as number of events versus number of eyes and percentages with 95% confidence interval in parentheses. n/a = not applicable.

**Table 4:** The effect of selective intra-arterial chemotherapy treatment on eye volume.

Dependent	Predictors	*p* Value
Healthy-controls vs. Rb-SIAC treated with SIAC ≤ 12 months of age	Age at scan in months	0.45
	Eye volume	<0.001
Healthy-controls vs. Rb-SIAC treated with SIAC > 12 months of age	Age at scan in months	<0.001
	Eye volume	<0.001
Rb-controls vs. Rb-SIAC that were treated with SIAC ≤ 12 months of age	Age at scan in months	0.66
	Eye volume	<0.001
Rb-controls vs. Rb-SIAC that were treated with SIAC > 12 months of age	Age at scan in months	<0.001
	Eye volume	0.007
Rb-SIAC that were treated with SIAC ≤ 12 months of age vs. Rb-SIAC that were treated with SIAC > 12 months of age	Age at scan in months	<0.001
	Number of SIAC cycles	0.06
	Eye volume	<0.001

Note.- Five multivariable logistic regression (forward Wald) between groups with age at scan in months, number of SIAC cycles, and eye volume as predictors with their respective *p* values. SIAC = selective intra-arterial chemotherapy; Rb-SIAC = retinoblastoma eyes that were treated with SIAC, Rb-control = retinoblastoma eyes that were treated with other eye-preserving treatment than SIAC; Healthy-controls = contralateral unaffected healthy eyes.

## Data Availability

The data that support the findings of this study are not openly available due to reasons of sensitivity and are available from the corresponding author upon reasonable request, only if permission of the providing center is obtained.

## Supplementary Materials

The following supporting information can be downloaded at: https://www.mdpi.com/article/10.3390/cancers16101899/s1, Supplement A: extracting eye volumes through MV-CNN; Figure S1: Intraclass correlation coefficient of manual volume versus MV-CNN generated volume in mm^3^.; Figure S2. Individual volume change per eye before and after treatment for the healthy-controls, Rb-controls, and Rb-SIAC; Table S1: Imaging features selected for scoring.; Table S2: Quantitative measurements by radiologists.; Table S3: Mean results for the ten-fold cross-validation for each included country and total.; Table S4: Characteristics for each group for the quantitative ocular volume measurements.; Table S5: Multivariable linear regression models of eye growth within the main groups with center and gender as potential predictors and their respective *p* values.
