# Supplementary material for: MR Imaging of Adverse Effects and Ocular Growth Decline after Selective Intra-Arterial Chemotherapy for Retinoblastoma
Source: Cancers (Basel). 2024 May 16;16(10):1899. doi: 10.3390/cancers16101899 (PMC11120425; doi:10.3390/cancers16101899)
Supplement: Supplementary file 1 [file cancers-16-01899-s001.zip › cancers-2981297-supplementary.pdf]

Supplementary Materials

Image preprocessing

Prior to automatic segmentation, preprocessing procedures were applied using tools from the FMRIB Software Library (FSL; https://fsl.fmrib.ox.ac.uk/) and the Insight Toolkit (ITK; https://itk.org/). First, a 3D Hough filter, implemented in ITK, was applied to the scans to construct a rough outline of each eye. These outlines, extrapolated by a radius of 6 mm, were converted into binary masks which were used as a region of interest for segmentation. The scans and masks were resampled to obtain equal voxel spacing of 0.29x0.29x2.1 mm using spline and nearest-neighbor interpolation, respectively. At last, the intensities of each scan were re-scaled in order that image intensities had a mean and variance of 0 and 1, respectively, within the union of the 6-mm mask of the eye.

MV-CNN Training

Based on our previously published model for ocular structure segmentation, eye globe segmentation was performed using the MV-CNN network with two pyramid scales. 2D patches of 32x32 pixels were extracted from the orthogonal planes of each 3D T1c scan (axial, coronal, sagittal). These patches were the input to each branch of the MV-CNN network. Target labels were mapped to binary outputs (0 for background and 1 for eye structure). The network was implemented in Python 3.6.9 using the framework of Tensorflow version 2.2.0 and trained on a NVIDIA GeForce GTX 1080 TI graphics processor unit (GPU) using CUDA 10.1 for accelerated training. Ten-fold cross-validation was applied to train and validate the network. For each fold, a network was trained on 90% of the training dataset and validated on the remaining 10%. The batch size was set to 64 and each network was trained for up to three epochs by minimizing a cross-entropy loss using ADAM optimizer. Dropout with ratio 0.25 was enabled to improve generalization capability of the networks and to prevent overfitting. A random subset of 20% of all training voxels was sampled to reduce computational demand and random reshuffling of the samples was done to allow for varied training. The evaluation metrics (Dice Similarity Coefficient [DSC], Intraclass correlation coefficient [ICC] and relative volume) of all ten folds were averaged to represent the overall performance of the network (see Table S3 and Figure S1. After ten-fold cross-validation, the MV-CNN network was retrained on the entire training dataset to maximize the amount of data seen by the network during training. This final model was eventually applied to the entire dataset to quantify the eye volumes.

Figure S1: Intraclass correlation coefficient of manual volume versus MV-CNN generated volume in mm³.

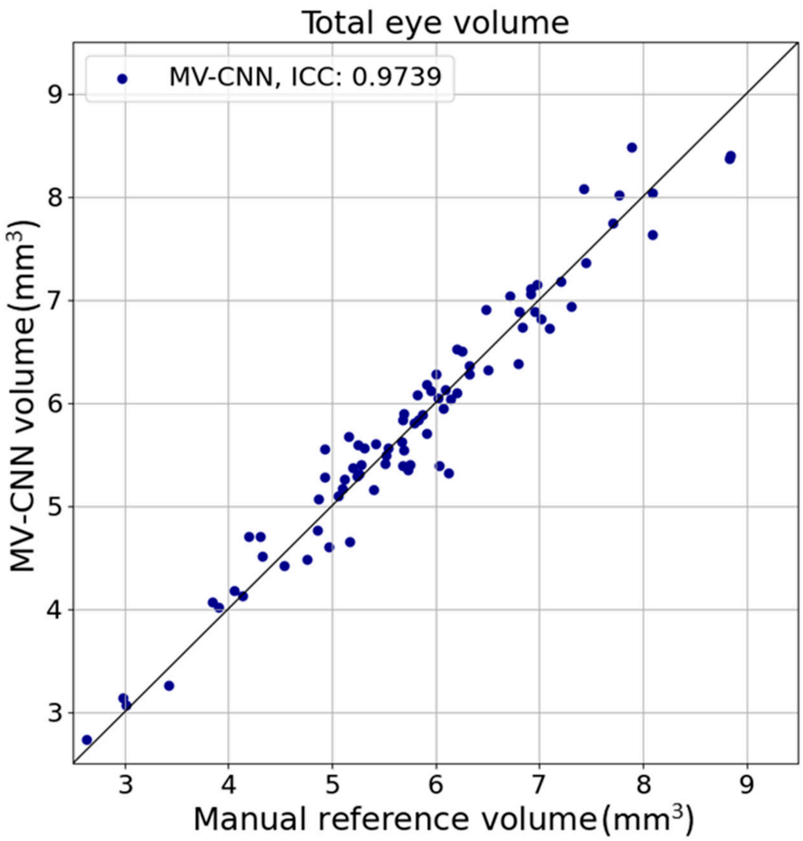

Note.- ICC = Intraclass correlation coefficient

Table S1: Imaging features selected for scoring.

Ocular	
1	Retinal detachment pretreatment*
2	Retinal detachment posttreatment*
3	Subretinal hemorrhage pretreatment*
4	Subretinal hemorrhage posttreatment*
5	Vitreous hemorrhage pretreatment*
6	Vitreous hemorrhage posttreatment*
7	Enhancement of the anterior eye segment*
8	Choroidal vascular infarction*
9	Choroidal thickening*
10	OD pretreatment axial length in mm†
11	OS pretreatment axial length in mm†
12	OD posttreatment axial length in mm†
13	OS posttreatment axial length in mm†
**Orbital (Preseptal space)**	
14	Palpebral inflammation (cellulitis)*
**Orbital (Prostseptal space)**	
15	Extraocular muscle fibrosis*
16	Extraocular muscle inflammation (myositis)*
17	Orbital fat enhancement (cellulitis)*
18	Orbital fat necrosis*
19	Optic nerve enhancement*
20	Perineural fibrosis optic nerve*
21	OD pretreatment optic nerve diameter 3 mm posterior to lamina cribrosa
22	OS pretreatment optic nerve diameter 3 mm posterior to lamina cribrosa
23	OD posttreatment optic nerve diameter 3 mm posterior to lamina cribrosa
24	OS posttreatment optic nerve diameter 3 mm posterior to lamina cribrosa
**Intracranial**	
25	Cerebral infarction*

Note.- *Features appearances were answered by yes or no. † measurements are performed in accordance with previous published work.[28] OD = right eye; OS = left eye

Table S2: Quantitative measurements by radiologists.

Feature	Rb-SIAC	Rb-controls	Healthy-controls	*p* value Rb-SIAC vs Rb-controls	*p* value Rb-SIAC vs Healthy-controls	*p* value Rb-controls vs Healthy-controls
**Pretreatment scan**						
**Optic nerve diameter in mm (SD)**	2.78 (0.37), n=160	2.74 (0.39), n=61	2.87 (0.32), n=86	0.43	0.02	0.01

**Axial eye length in mm (SD)**	20.31 (1.28), n=181	20.37 (1.22), n=52	20.98 (1.06), n=99	0.83	<0.001	0.001
**Posttreatment scan**						
**Optic nerve diameter in mm (SD)**	2.72 (0.42), n=226	2.87 (0.34), n=66	2.98 (0.31), n=122	0.02	<0.001	0.009
**Axial eye length in mm (SD)**	20.49 (1.34), n=234	21.11 (1.05), n=67	21.53 (1.10), n=126	0.001	<0.001	0.003
**Difference between mean post- and pretreatment scans**						
**Optic nerve diameter in mm (SD)**	-0.11 (0.34), n=158	0,09 (0,20), n=42	0.07 (0.18), n=85	<0.001	<0.001	0.55
**Axial eye length in mm (SD)**	0.12 (1.04), n=184	0.71 (0.94), n=45	0.50 (0.67), n=99	<0.001	<0.001	0.53

Note.- Data presented as mean measurement in mm and standard deviation in parentheses. Optic nerve diameter measurements were performed at 3 mm posterior to the lamina cribrosa. *p* values based on Mann-Whitney *U*-test.

Table S3: Mean results for the ten-fold cross-validation for each included country and total.

Dataset:	Dice Similarity Coefficient mean (SD)	Mean relative volume (SD)
**Lausanne**	0.964 (± 0.013)	0.997 (± 0.048)
**Siena**	0.950 (± 0.016)	1.01 (± 0.058)
**Amsterdam**	0.954 (± 0.013)	1.006 (± 0.46)
**Total**	0.954 (± 0.014)	1.005 (± 0.049)

Note.- Data presented as mean Dice Similarity Coefficient (standard deviation [SD]) and standard deviation in parentheses or as mean relative volume in mm^3 and standard deviation in parentheses.

Table S4: Characteristics for each group for the quantitative ocular volume measurements.

	Group (n= number of unique eyes)	Rb-SIAC (n=198)	Rb-controls (n=65)	Healthy-controls (n=155)	Total (n=418)
**Pretreatment**	**Eyes**	138	39	119	296
	**Median age at scan [IQR], (range)**	16 [9-31], (1-155)	9 [8-19], (1-44)	25 [13-39], (0-155)	18 [10-33], (0-155)
	**Center**				
	Lausanne n, %	69 (50)	26 (67)	26 (22)	121 (41)
	Siena n, %	60 (43)	10 (26)	36 (30)	106 (36)
	Amsterdam n, %	9 (7)	3 (8)	57 (48)	69 (23)
**Posttreatment**	**Eyes**	197	58	97	353
	**Median age at scan [IQR], (range)**	28 [18-45], (3-159)	24 [19-35], (3-127)	36 [21-54], (6-159)	28 [19-45], (3-159)
	**Center**				
	Lausanne n, %	127 (64)	46 (79)	57 (59)	230 (65)

	Siena n, %	60 (30)	12 (21)	37 (38)	110 (31)
	Amsterdam n, %	10 (5)	0	3 (3)	13 (4)
	**Median number of SIAC cycles [IQR], (range)**	3, [2-5], (1-9)	n/a	n/a	3, [2-5], (1-9)
	Median number of SIAC cycles for eyes that received SIAC ≤ 12 months of age*	3, [2-6], (1-9)	n/a	n/a	3, [2-6], (1-9)
	Median number of SIAC cycles for eyes that received SIAC > 12 months of age*	3, [2-5], (1-9)	n/a	n/a	3, [2-5], (1-9)

Note.- Data presented as number of eyes with percentages in parentheses or median age in months [interquartile range], (range). The total percentages might be lower or higher than 100 due to rounding of the numbers. n/a = not applicable.

* Both groups, median number of SIAC cycles for eyes that received SIAC ≤ 12 months of age and > 12 months of age, were compared to each other through Mann-Whitney *U*-test (*p*=0.75).

Table S5: Multivariable linear regression models of eye growth within the main groups with center and gender as potential predictors and their respective *p* values.

	Predictors	***p* value**
**Pretreatment**	Healthy-controls vs retinoblastoma (Rb-controls and Rb-SIAC)	<0.001
	Age	<0.001
	Center	0.031
	Gender	0.11
**Pretreatment**	Rb-SIAC vs Rb-controls	0.039
	Age	<0.001
	Center	0.023
	Gender	0.46
**Posttreatment**	Healthy-controls vs Rb-controls	0.062
	Age	<0.001
	Center	0.46
	Gender	0.16
**Posttreatment**	Healthy-controls vs Rb-SIAC	<0.001
	Age	<0.001
	Center	0.56
	Gender	0.13
**Posttreatment**	Rb-SIAC vs Rb-controls	<0.001
	Age	<0.001
	Center	0.31
	Gender	0.073

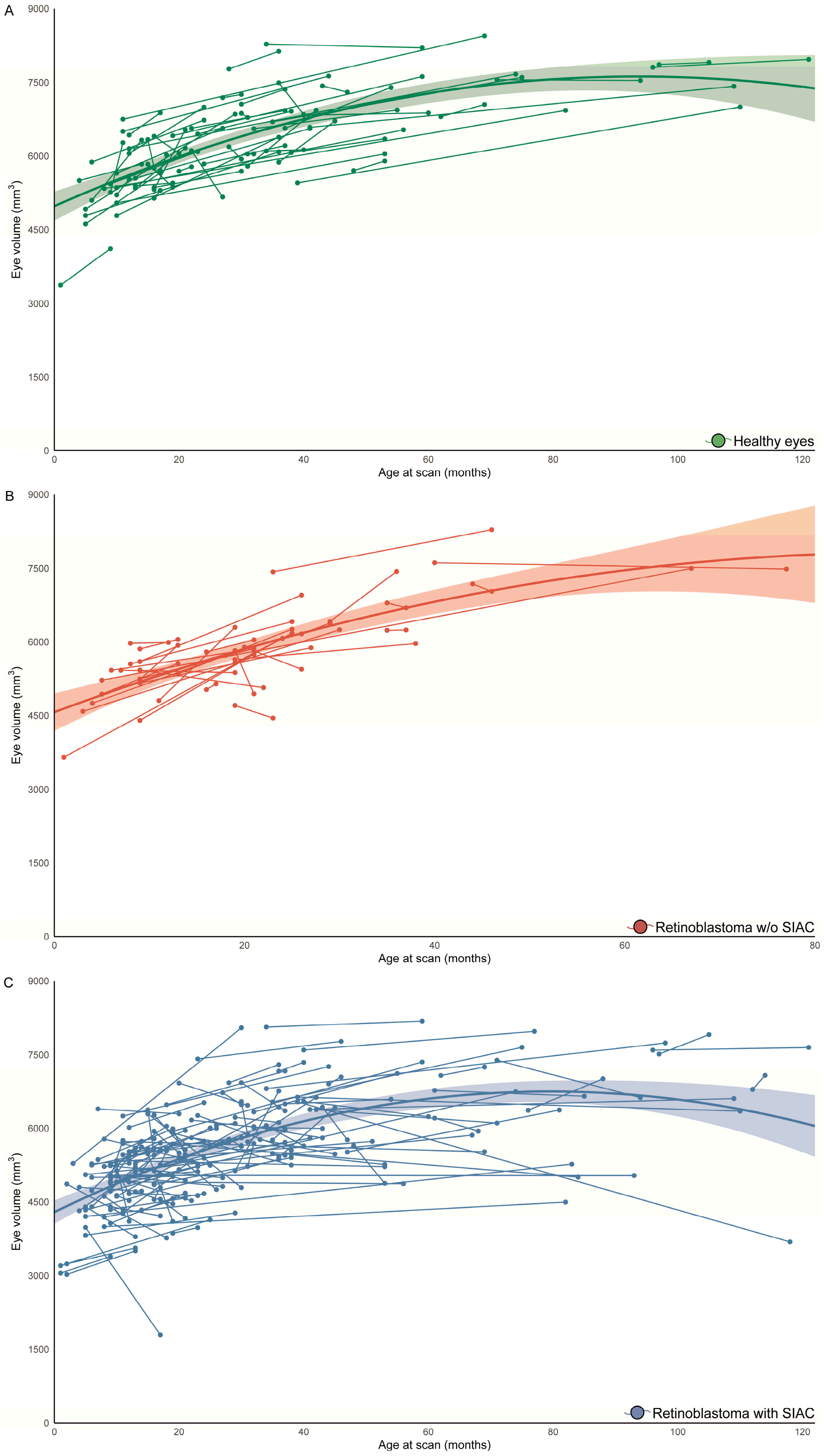

A
B
C
Eye volume (mm^3)
Age at scan (months)
Healthy eyes
Retinoblastoma w/o SIAC
Retinoblastoma with SIAC
9000
7500
6000
4500
3000
1500
0
20
40
60
80
100
120

Figure S2. Individual volume change per eye before and after treatment for the healthy-controls, Rb-controls and Rb-SIAC.

A) Individual volume change per eye before and after treatment for the healthy eyes (healthy-controls) with the mean volume change per eye and 95% confidence interval. B) Individual volume change per eye before and after treatment for the retinoblastoma eyes treated without SIAC (Rb-controls) with the mean volume change per eye and 95% confidence interval. C) Individual volume change per eye before and after treatment for retinoblastoma eyes treated with SIAC (Rb-SIAC) with the mean volume change per eye and 95% confidence interval. Note.- Lines between the dots are individual trajectories for each eye. SIAC = selective intra-arterial chemotherapy.
